# The Vaginal-PVPA: A Vaginal Mucosa-Mimicking In Vitro Permeation Tool for Evaluation of Mucoadhesive Formulations

**DOI:** 10.3390/pharmaceutics12060568

**Published:** 2020-06-19

**Authors:** Margherita Falavigna, Martina Pattacini, Richard Wibel, Fabio Sonvico, Natasa Škalko-Basnet, Gøril Eide Flaten

**Affiliations:** 1 Drug Transport and Delivery Research Group, Department of Pharmacy, UiT The Arctic University of Norway, Universitetsvegen 57, 9037 Tromsø, Norway; martina.pattacini@studenti.unipr.it (M.P.); richard.wibel@yahoo.de (R.W.); natasa.skalko-basnet@uit.no (N.Š.-B.); 2 Dipartimento di Scienze degli Alimenti e del Farmaco, Università di Parma, Parco Area delle Scienze 27/a, 43124 Parma, Italy; fabio.sonvico@unipr.it

**Keywords:** vaginal drug administration, chitosan-coated liposomes, mucoadhesion, permeation, in vitro studies, simulated vaginal fluid, pH-dependent drug permeation, mucus

## Abstract

Drug administration to the vaginal site has gained increasing attention in past decades, highlighting the need for reliable in vitro methods to assess the performance of novel formulations. To optimize formulations destined for the vaginal site, it is important to evaluate the drug retention within the vagina as well as its permeation across the mucosa, particularly in the presence of vaginal fluids. Herewith, the vaginal-PVPA (Phospholipid Vesicle-based Permeation Assay) in vitro permeability model was validated as a tool to evaluate the permeation of the anti-inflammatory drug ibuprofen from liposomal formulations (i.e., plain and chitosan-coated liposomes). Drug permeation was assessed in the presence and absence of mucus and simulated vaginal fluid (SVF) at pH conditions mimicking both the healthy vaginal premenopausal conditions and vaginal infection/pre-puberty/post-menopause state. The permeation of ibuprofen proved to depend on the type of formulation (i.e., chitosan-coated liposomes exhibited lower drug permeation), the mucoadhesive formulation properties and pH condition. This study highlights both the importance of mucus and SVF in the vaginal model to better understand and predict the in vivo performance of formulations destined for vaginal administration, and the suitability of the vaginal-PVPA model for such investigations.

## 1. Introduction

The administration of drugs to the vaginal site has proven advantageous for both local and systemic therapy thanks to the possibility of self-administration, the potential of prolonged treatment due to long residence time of the drug *in loco*, and the avoidance of first-pass metabolism in the case of systemic therapies [1]. Research efforts have been directed towards the development of drug formulations for treating vaginal infections, sexually transmitted diseases, cervical cancer, and hormonal contraception [2]. During the development process of drug formulations destined to the vaginal site, it is of utmost importance to take into consideration the challenges related to this site of administration, which are mainly associated with the vaginal anatomy and its secretions [3]. For instance, the mixture of fluids that end up in the vagina (i.e., cervical mucus, semen, etc.) and those that originate from the vagina can greatly impact the retention of the formulation and the subsequent drug release [4]. This mixture of fluids is, in fact, the first physical barrier that the administered formulations encounter, and it has been demonstrated that its composition, osmolarity, and pH can significantly affect the delivery of drugs [5]. In light of the characteristics of these fluids and their impact on the efficacy of the drug formulation, it is possible to develop drug-delivery systems able to exploit the nature of the vaginal environment for a better drug therapy. For example, the preparation of mucoadhesive systems has been established as one of the strategies able to improve the residence time of the formulation in the vagina. Especially, chitosan-based products have gained increasing momentum due to their biodegradability, biocompatibility, mucoadhesion, and intrinsic antimicrobial effects [6].

A general consensus has been reached in terms of how drug formulation development should be carried out, and a special focus has been put on the efforts that the scientific community should make in terms of the replacement, reduction, and refinement of animal research [7]. As a result, the past decades witnessed an increasing need for in vitro models, including ones mimicking the vaginal site. In comparison to models mimicking the intestinal and ophthalmic site, relatively limited numbers of models are available for reliable prediction of vaginal drug permeation. So far, the in vitro models have been developed to predict (i) drug permeability in the vaginal environment (i.e., cell-based models, [2]) and (ii) to assess the mucoadhesive potential of the formulation in the vagina [5,8]. Moreover, efforts have been devoted to mimicking the fluids present in the vagina, such as the simulated vaginal fluid (SVF) proposed by Owen and Katz [9], which has been largely used to simulate the composition of vaginal fluids and test the diffusion and permeation of novel drug-delivery systems [5,6,10]. Even though the above-mentioned in vitro models and simulated fluid serve as great tools in developing formulations for vaginal administration, the lack of a reproducible, artificial, simple, and high throughput in vitro permeation model closely mimicking the vaginal mucosa and its environment was the reason for modifying the already established mucus-PVPA (Phospholipid Vesicle-based Permeation Assay) in vitro permeability model [11] to closely mimic these conditions. We have shown earlier that the mucus-PVPA model is a reliable and predictive artificial permeation tool able to mimic intestinal mucosal barriers and aid in assessing mucosal drug permeability [11,12]. Moreover, the original PVPA barriers were previously used to study in vitro drug permeation from mucoadhesive formulations destined to the vaginal site [13]. In the current study, we went a step further and developed a novel vaginal model including both the mucus layer as well as SVF. We applied, as a model drug, the anti-inflammatory drug ibuprofen in different formulations, namely solution as well as liposomal and mucoadhesive formulation, and we studied its permeability by employing the developed vaginal model. To address the challenges of vaginal drug delivery, we evaluated drug permeation at two pH conditions: the healthy vaginal conditions of pre-menopausal women (pH of ≈ 4.5) and the conditions caused by vaginal infections as well as conditions present in pre-puberty or post-menopause women (pH ≈ 7–8; [14,15]). In the present study, we were able to prove that the formulation, pH, and presence of mucus and SVF affect the penetration potential of a model drug across the PVPA barriers, and the obtained results suggest that the vaginal-PVPA could be a reliable in vitro tool for the development of drug-delivery systems destined for vaginal administration.

## 2. Materials and Methods

### 2.1. Materials

E80 egg phospholipids (80% phosphatidylcholine) and S100 soybean lecithin lipids (>94% phosphatidylcholine) were obtained from Lipoid GmbH (Ludwigshafen, Germany). Acetic acid, bovine serum albumin, calcium hydroxide, chitosan (low molecular weight, 75–85% deacetylated), chloroform, D(+)-glucose, glycerol solution, ibuprofen, lactic acid, mucin from porcine stomach type III, potassium phosphate monobasic, propylene glycol, sodium chloride, sodium hydroxide, and sodium phosphate dibasic dodecahydrate were products of Sigma Aldrich Chemie GmbH (Steinheim, Germany). Ethanol, methanol, and acetic acid were obtained from VWR Chemicals (Fontenaysous-Bois, France). Potassium hydroxide was obtained from Norsk Medisinaldepot (Oslo, Norway).

### 2.2. Preparation of Plain Liposomes

Plain liposomes were prepared by using the thin-film hydration method described earlier by Berginc et al. [16]. Briefly, 200 mg of S100 soybean lecithin lipids and 20 mg of ibuprofen were dissolved in a mix of methanol and chloroform (2:4 *v*/*v*). The solvent was evaporated using a Büchi rotavapor with a vacuum controller (Büchi Labortechnik, Flawil, Switzerland) for a total of 3 h at 60 mBar and 45 °C. The resulting thin film was hydrated using 10 mL of distilled water, and the liposomal dispersion was manually mixed until a homogeneous liposomal suspension was obtained. The liposomal formulation was stored in the refrigerator (4–6 °C) overnight prior to a size reduction. The size of the liposomes was reduced stepwise by extruding through polycarbonate membranes with pore sizes of 0.8, 0.4, and 0.2 µm (Nuclepore Track-Etch Membran, Whatman House, Maidstone, UK). Four extrusion cycles were carried out for each pore size. As a control, liposomes without drugs were also prepared in the same manner (i.e., empty plain liposomes).

### 2.3. Entrapment Efficiency and Recovery

The amount of unentrapped ibuprofen was separated from the one encapsulated in the liposomes via dialysis following the method described by Falavigna et al. [11]. Plain liposomes (4 mL) were placed into a dialysis tubing with a molecular weight cut-off of 12,000–14,000 Da (Medicell International Ltd., London, UK) and were dialyzed against distilled water (1 L) for a total of 4 h at room temperature (23–25 °C). The chosen volume of the dialysis medium assured the solubilization of the drug. To free the ibuprofen encapsulated in the liposomes, aliquots of the plain liposomes were dissolved using methanol. The amount of the encapsulated drug was compared with the amount in the dialysis medium (unentrapped ibuprofen) and with the amount initially loaded into the formulation in order to calculate entrapment efficiency (EE %) and recovery (%). Ibuprofen was quantified via High Performance Liquid Chromatography (HPLC) using a Waters 2690 Separation Module HPLC system, equipped with Waters 996 Photodiode Array Detector (Waters Corporation, Milford, MA, USA), and a Symmetry C18 3.9 × 150 mm column (Waters, Milford, MA, USA). The mobile phase was composed of 25% acetic acid solution 0.1% *v*/*v* and 75% methanol with a flow rate of 0.5 mL/min and at 220 nm (retention time 7.5 min) (linearity range: 5–500 nmol/mL, R^2^ 0.999).

(1) $$EE\left(\%\right)=\frac{Amount\;of\;encapsulated\;drug}{Amount\;of\;drug\;initially\;loaded\;}\times 100$$

(2) $$Recovery\;\left(\%\right)=\frac{Amount\;of\;encapsulated\;drug+Amount\;of\;unentrapped\;drug}{Amount\;of\;drug\;initially\;loaded\;}\times 100$$

### 2.4. Preparation of Chitosan-Coated Liposomes

Plain liposomes were coated in the absence of unentrapped drug using a 0.1% (*w*/*v*) chitosan solution prepared in 0.1% (*v*/*v*) glacial acetic acid following the method described by Jøraholmen et al. [17]. The chitosan solution was added dropwise to an equal volume of plain liposomes under controlled magnetic stirring. The mix was left to stir for 2 h under magnetic stirring at room temperature (23–25 °C). The chitosan-coated liposomes (coated liposomes) were then stored in the refrigerator (4–6 °C) for at least 3 h prior to further use. As a control, liposomes without the drug were also prepared in the same manner (i.e., empty coated liposomes).

### 2.5. Size and Zeta Potential Measurements

The size and zeta potential of plain and coated liposomes was determined with the use of a Malvern Zetasizer Nano ZS (Malvern, Oxford, UK) following the method described by Falavigna et al. [11]. Size measurements were carried out in a polystyrene cuvette, where the sample was diluted 1:100 (*v*/*v*) with distilled water. Zeta potential measurements were performed in a folded capillary cell (DTS1070, Malvern, Malvern, Oxford, UK), where the sample was diluted 1:30 (*v*/*v*) in filtered tap water. Two replicates for each batch (three batches for each liposomal formulation) were analyzed at room temperature (23–25 °C) for both zeta potential and size measurement.

### 2.6. PVPA Barriers Preparation

The preparation of the PVPA barriers was carried out following the method previously described by our group [11,18]. Briefly, liposomes with two different size distributions (0.4 and 0.8 µm) were prepared from E80 egg phospholipids (80% phosphatidylcholine), and immobilized in and on top of nitrocellulose membrane filters (pore size 0.65 µm, Millipore, Billerica, MA, USA), which were fused to Transwell inserts (surface area 0.33 cm^2^, Corning Inc., Corning, NY, USA). Cycles of centrifugation, thawing and freezing permitted the completion of the barriers. The size of the liposomes prior to their immobilization in and on top of the filters was carried out according to the method described in Section 2.5.

### 2.7. Preparation of Mucus and Simulated Vaginal Fluid

The mucus layer was obtained as previously described [12] by hydrating mucin from porcine stomach type III with phosphate buffer saline (PBS) pH 4.6 or 7.0 in order to obtain a final concentration of 10 mg/mL.

Simulated vaginal fluid (SVF) was prepared using the method described by Owen and Katz [9] with the composition shown in Table 1 in order to obtain a final pH of 4.6. The SVF was adjusted to pH 7.0 to simulate the environment found in the case of vaginal infection, pre-puberty, or post-menopause [14,15]. Once prepared, SVF was stored at room temperature (23–25 °C) and used within one week.

### 2.8. In Vitro Permeability Study Using the PVPA Barriers

The compatibility of the PVPA barriers with different setups (Table 2) was investigated by determining the permeability of the highly hydrophilic marker calcein (5 mM, pH 4.6) and by measuring the electrical resistance across the barriers after 5 h of the permeation experiment at room temperature (23–25 °C), as previously described [12]. The investigated setups consisted of setup 1, setup 2, and setup 3. Setup 1 included naked PVPA barriers, setup 2 had an addition of mucus at two different pH conditions on top of the PVPA barriers (setup 2A and B), and setup 3 involved the addition of mucus + SVF at two different pH conditions (Setup 3A and B) (Table 2). For Setups 2 and 3, mucus (50 µL) or mucus + SVF (50 µL + 10 µL) was placed on top of the PVPA barriers 5 min prior to the start of the permeation experiment. For setup 3, SVF was placed on top of the mucus layer, and mucus and SVF were used at the same pH.

Once the maintained integrity of the PVPA barriers in the different setups (Table 2) was confirmed, the permeability of ibuprofen from plain liposomes, coated liposomes, or ibuprofen solution (ibuprofen in 50% *v*/*v* propylene glycol, control solution) was determined after 5 h of drug permeation, as previously described [11,18]. The ibuprofen formulations were placed (100 µL) in the donor compartment on top of the PVPA barriers for each setup. Plain liposomes (2 mg/mL) were diluted 1:2 (*v*/*v*) with distilled water to obtain the same ibuprofen concentration as the coated liposomes and as the ibuprofen solution formulations (i.e., 1 mg/mL). This allowed the comparison of the cumulative amount of drug permeated over time.

The permeability experiment was commenced by placing the Transwell inserts containing the PVPA barriers loaded with the desired setup and the investigated formulation (i.e., plain liposomes, coated liposomes, or ibuprofen solution) in an acceptor compartment containing PBS pH 7.4 (600 µL) simulating the systemic blood circulation. The barriers were moved to new acceptor compartments containing fresh PBS pH 7.4 after 1, 2, 3, 3.5, 4, 4.5, and 5 h to preserve sink conditions. After 5 h, the electrical resistance across the barriers was measured to confirm the correct functionality of the barriers. Samples from the donor (10 µL) and the acceptor (200 µL) compartment were withdrawn to quantify the amount of ibuprofen left in the donor and permeated through the barriers after 5 h, respectively. The sample from the donor compartment was diluted in methanol, and ibuprofen was quantified by following the method described in Section 2.3. The amount of ibuprofen permeated through the barriers into the acceptor compartment was quantified at 220 nm using a Spark Multimode Microplate Reader (Tecan, Männendorf, Switzerland) (linearity range: 2–200 nmol/mL, R^2^ 0.999). The apparent permeability coefficient (P_app_, cm/s) of ibuprofen was calculated using the following equation.

(3) $$P_{app}\left(\frac{cm}{s}\right)=\frac{dQ}{dt}\times \frac{1}{A\times Cd}$$

where *dQ*/*dt* represents the slope at the steady state conditions (nmol/s), *A* is the surface area of the barriers (0.33 cm^2^), and *Cd* is the concentration of ibuprofen in the donor (nmol/mL). For each setup and each formulation, 6 PVPA barriers were used and three replicates of the same experiment were performed (n = 18).

### 2.9. Mucoadhesive Properties of Coated Liposomes

The mucoadhesive properties of the liposomes were tested by measuring their in vitro binding to mucin from porcine stomach type III by utilizing the method that Jøraholmen et al. described [19]. Briefly, 1 mL of liposomes (either plain or coated) were placed on top of the same volume of mucus (i.e., mucin 10 mg/mL, either pH 4.6 or 7.0). The mix was left to incubate at room temperature (23–25 °C) for 2 h, which was followed by ultra-centrifugation at 10 °C for 1 h using an Optima LE-80 (Beckman Instruments, Palo Alto, CA, USA) with a speed of 216,000× *g*. The amount of mucin left in the supernatant was quantified using a Spark Multimode Microplate Reader (Tecan, Männendorf, Switzerland) at 251 nm (linearity range: 0.05–0.5 mg/mL, R^2^ 0.999). The mucin-binding efficiency was determined by following the method described by Naderkhani et al. [13]. The mucin-binding efficiency of the coated liposomes was compared to the plain liposomes, together with the comparison of the mucin-binding efficiency of the formulations at pH 4.6 and 7.0. Each sample was tested in triplicate at each pH.

### 2.10. Statistical Evaluation

The statistical evaluation of the obtained results was carried out using GraphPad prims 8.0 software. Student *t*-test was used to highlight significant differences (*p* < 0.05) between two sets of data. A comparison between three or more sets of data was performed using one-way ANOVA, and a significant difference (*p* < 0.05) was determined using the Bonferroni multiple comparison *post hoc* test.

## 3. Results and Discussion

During the development and optimization of new drug formulations, it is important to study their in vitro behavior as well as penetration potential across biological barriers before proceeding to in vivo or clinical testing [20]. Moreover, the use of animal tissues [21,22] during in vitro testing can be substituted by the use of artificial membranes to better comply with the need of replacement and reduction of animal use for research purposes [7]. For this reason, we aimed at further developing the mucus-PVPA model [11] to specifically mimic the vaginal environment. The development of such in vitro model, comprising an artificial barrier, would allow a fast and precise assessment of drug permeation from formulations destined for vaginal administration. To investigate the model’s ability to handle relevant vaginal formulations, liposomal formulations able to be retained at the vaginal site and able to release the incorporated drug to the mucosal tissue were used as simple model formulations.

### 3.1. Validation of the Vaginal-PVPA Model in Terms of Barrier Integrity

**Figure 1:**
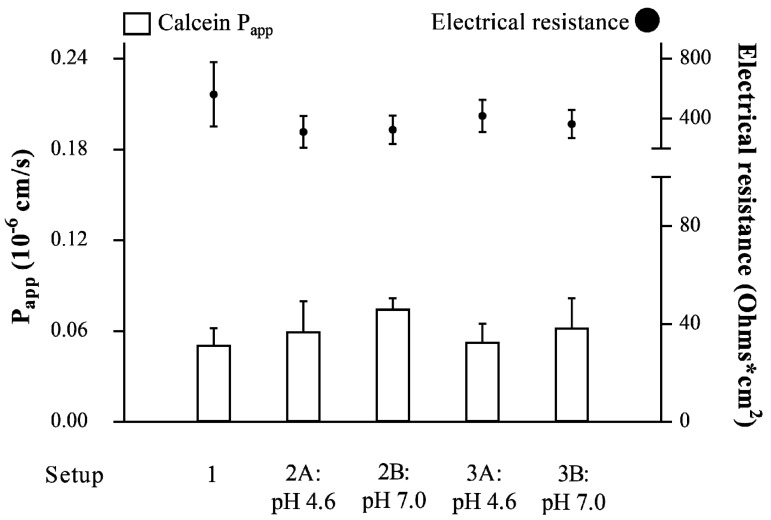
Apparent permeability (P_app_) of calcein (left y-axis) and electrical resistance across the Phospholipid Vesicle-based Permeation Assay (PVPA) barriers (right y-axis) in the presence of different setups utilized in the study (mean ± SD, n = 18). Setup 1 (i.e., naked barriers), Setup 2A and B (i.e., mucus pH 4.6 or 7.0), and Setup 3A and B (i.e., mucus + SVF pH 4.6 or 7.0).

To develop an in vitro permeability model able to mimic the vaginal environment, it is crucial to include the presence of a mucus layer, SVF, and relevant pH conditions on top of an artificial permeation barrier. Thus, the PVPA barriers were combined with a mucus layer and SVF. To ensure proper assembly of the PVPA barrier, the size distribution of the liposomes immobilized in the PVPA barrier filters and on top of the filters was evaluated. The liposomes inside the barriers had a size distribution around 500 nm (519 ± 57 nm), whereas the liposomes used on top of the barriers were around 700 nm (735 ± 50 nm). Moreover, to assure the barriers’ correct functionality during permeation experiments, the integrity of the PVPA barriers was studied in the presence and absence of mucus, and the combination of mucus + SVF at pH 4.6 and 7.0 (Table 2) to mimic different vaginal pH conditions. This assessment was carried out by measuring the P_app_ of the highly hydrophilic marker calcein and the electrical resistance across the PVPA barriers at the end of the permeability experiment. Calcein permeability values below 0.06 10^−6^ cm/s and electrical resistance above 290 Ohm cm^2^ indicate barrier integrity, as previously demonstrated [11,12]. As observed in Figure 1, the PVPA barriers maintained their integrity in all proposed setups, which indicated that the presence of mucus and mucus + SVF did not impair the functionality of the permeation barriers at the tested pH conditions. In virtue of these results, all setups were employed to study the permeability of ibuprofen from different formulations (i.e., ibuprofen solution, plain liposomes, and coated liposomes), and the impact that the presence of mucus and SVF has on drug permeation was evaluated.

Moreover, the morphologic examination of the PVPA barriers in the presence and absence of mucus had already been carried out in our previous study [11] and the results indicated that there was no morphological change in the barriers between the presence and absence of the mucus layer. In the present study, the mucus layer was the component closely in contact with the PVPA barriers and the one that was in the highest volume (50 µL) compared to the SVF (10 µL, placed on top of the mucus layer). For this reason, we do not think that SFV would have had an impact on the morphology and connected integrity of the PVPA barriers. If SVF would have impacted the morphology of the barriers, its effect would have been translated in a change of calcein permeability and electrical resistance across the barriers. As can be seen in Figure 1, both calcein P_app_ and electrical resistance did not change for all setups.

### 3.2. Characterization of the Liposomal Formulations

The use of liposomes as drug-delivery systems for vaginal administration has gained increasing attention over the past decades [17,23,24,25] due to their biodegradability, biocompatibility, non-irritating properties towards the vaginal mucosa and to their intrinsic capacity of shielding active substances from the possible degradation occurring at the vaginal site [26]. Moreover, the coating of these formulations with chitosan can improve the mucoadhesive and antimicrobial properties of such drug-delivery systems [6,13,17], which enables higher resident time in the vagina and aids against bacterial infections. In light of this, we prepared both non-coated liposomes (i.e., plain liposomes) and coated liposomes incorporating the anti-inflammatory lipophilic model drug ibuprofen (LogP 3.97, [27]) to evaluate their potential as delivery systems for vaginal drug administration utilizing the vaginal-PVPA model. When incorporating a drug in the liposomal structure, the physicochemical characteristics of the drug will dictate whether it will be placed in the aqueous core and/or in the phospholipid bilayer of the liposome [28]. The lipophilicity of ibuprofen strongly suggests that the drug would place itself in the phospholipid bilayer rather than in the aqueous core of the liposome. This evidence has already been proven in the literature [29].

Size and zeta potential of the drug-loaded liposomes were compared to the drug-free liposomes (i.e., empty liposomes). As observed in Table 3, the size of the liposomes was around 200 nm for plain liposomes (size suggested when aiming at vaginal drug delivery, [30]) and 300 nm for coated liposomes. Moreover, the polydispersity index suggests a homogeneous size distribution for plain liposomes and a more heterogeneous one for coated liposomes (Table 3). Both size and zeta potential increased from plain to coated liposomes, which is in agreement with the literature and proves that the coating took place [31,32]. The coating was especially evident for the liposomes containing the drug, where the size increased from around 200 to 300 nm and the zeta potential changed from −14 to +42–65 mV, respectively. The size and zeta potential difference between empty and drug-containing plain and coated liposomes could be due to the presence of ibuprofen (Table 1). In fact, the negative charge resulting from the carboxyl group of ibuprofen can interact with the positive charges provided by the amino groups of chitosan, resulting in a more pronounced coating effect compared to empty liposomes, and this led to a higher size and zeta potential. The entrapment efficiency was found to be high (91.06 ± 3.40%) for both plain and coated liposomes, as the coating was carried out in the absence of the unentrapped drug.

### 3.3. Dependence of Ibuprofen Permeability on the Formulation Type, Experimental Setup, and pH

Once the stability of the permeability model and the characterization of the liposomes had been assessed, the permeation of ibuprofen from solution, plain liposomes, and coated liposomes was evaluated at two pH conditions (i.e., pH 4.6 and 7.0) using different setups (Table 2) to evaluate if drug permeation would depend on (i) the specific formulation, (ii) the employed setup (i.e., the presence of SVF and/or mucus), and/or (iii) the pH. This investigation would allow the selection of the appropriate setup to be utilized for assessing drug permeation from formulations destined for vaginal administration and the estimation of the best formulation for such a drug administration route. This study also aimed at highlighting whether differences in the environmental pH would lead to variations in drug permeation. This was especially important since the vaginal pH can largely shift, according to various factors (e.g., age, bacterial infection, menstrual cycle, etc.), and can change from a slightly acidic pH in healthy conditions to a neutral one in case of vaginal infection, pre-puberty, or post-menopause [14,15].

**Figure 2:**
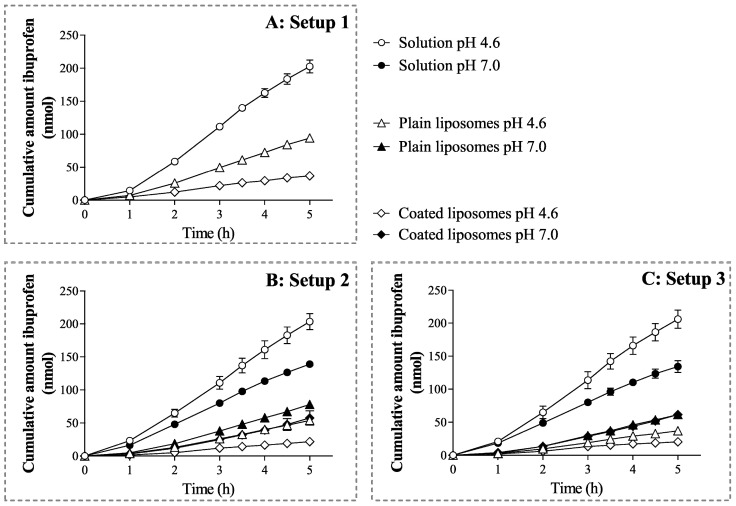
Cumulative amount of ibuprofen permeated across the barriers using (**A**) Setup 1 (i.e., naked barriers), (**B**) Setup 2A and 2B (i.e., mucus pH 4.6 or 7.0), and (**C**) Setup 3A and 3B (i.e., mucus + simulated vaginal fluid (SVF) pH 4.6 or 7.0) from ibuprofen solution (circle symbol, o), plain liposomes (triangle symbol, ∆), or coated liposomes (diamond symbol, ◊). Empty symbols indicate pH 4.6 and full symbols indicate pH 7.0. (Mean ± SD, n = 18).

**Figure 3:**
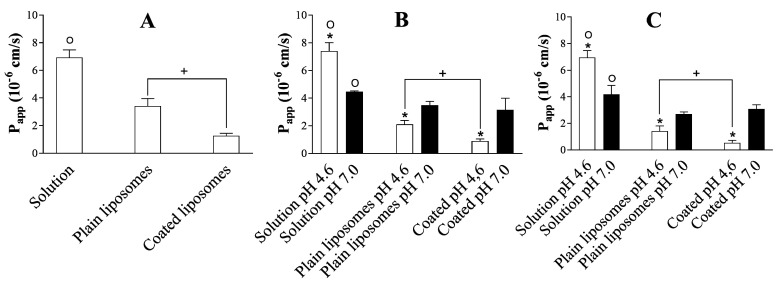
Apparent permeability (P_app_) of ibuprofen across the barriers from ibuprofen solution, plain liposomes, and coated liposomes using (**A**) Setup 1 (i.e., naked barriers), (**B**) Setup 2A and B (i.e., mucus pH of 4.6 or 7.0), and (**C**) Setup 3A and B (i.e., mucus + SVF pH of 4.6 or 7.0) (mean ± SD, n = 18).* Represents statistical difference in P_app_ between pH of 4.6 and 7.0 within the same formulation. ^o^ Represents statistical difference in P_app_ between ibuprofen solution and both liposomal formulation at the same pH. ^+^ Represents statistical difference in P_app_ between plain and coated liposomes at the same pH.

During the permeation experiments, the concentration of drugs in the donor compartment was the same for all formulations. Figure 2A and Figure 3A depict the cumulative amount of ibuprofen permeated over time across the PVPA barriers and the P_app_ of the drug, respectively, in the absence of mucus and SVF from the different formulations. Both cumulative amount of permeated drug and P_app_ were largely dependent on the specific formulation. In fact, both liposomal formulations exhibited a significantly lower drug permeation compared to the ibuprofen solution (Figure 2A and Figure 3A). This behaviour is expected, as liposomal formulations are known to provide a sustained release of the incorporated drug, which affects the amount of free drug available for permeation [23]. This is a very important feature when formulations aim at prolonging the drug release window at the vaginal site [17,25]. Moreover, the coated liposomes provided the lowest drug permeation (Figure 3A). This was likely due to factors such as (i) the interaction of the positively charged coated liposomes with the slightly negatively charged liposomes forming the PVPA barriers [33] and (ii) the electrostatic interaction that can occur between the negative charges resulting from the carboxyl group of ibuprofen and the positive charges provided by the amino groups of chitosan [34], thus resulting in the slower drug release and permeation.

When evaluating the setups where mucus was present on top of the PVPA barriers at pH 4.6 and 7.0 (Figure 2B and Figure 3B, Setup 2A and B), a similar trend was observed in terms of the permeation of ibuprofen from the solution and from the liposomal formulations. In fact, for both plain and coated liposomes, a lower amount of drug was able to permeate across the PVPA barriers when compared to the solution. The difference between solution and liposomal formulations is most likely due to the sustained release commonly seen for the drug from the liposomes. Moreover, in this setup, the pH of the mucus layer greatly affected the permeation of the drug, and the pH effect was also dependent on the type of formulation (Figure 2B and Figure 3B). For instance, the permeation of ibuprofen from the solution was higher at pH 4.6 when compared to pH 7.0 (Figure 3B). This result can be traced back to the degree of ionization that the drug has at the two pH conditions since the ionization of ibuprofen (pKa 4.45, [35]) can largely change from pH 4.6 to 7.0, and this can have an effect on the permeation of the drug itself. In fact, it has been previously shown that the permeation of ibuprofen across the PVPA barriers significantly decreases when going from an acidic to a neutral pH [12]. The same pH-dependent drug permeation was not found for the liposomal formulations. For both plain and coated liposomes, the permeation of the drug was found to be significantly lower at pH 4.6 compared to pH 7.0 (Figure 3B). This behaviour can be due to a different extent of diffusion of the liposomal formulations through the mucus layer or to a different extent of drug release at various pH conditions. In fact, the drug may reach the surface of the permeation barrier by (i) progressing through the mucus layer while remaining entrapped in the liposomes and/or (ii) by being released by the formulation and diffusing throughout the mucus layer as a free drug [36]. In this regard, Figure 3B shows that a different degree/mechanism of drug diffusion at the two pH conditions can be the reason for the difference in drug permeation. The diffusion of the formulation can both depend on its characteristics (i.e., size, surface charge, and geometry) [37] and the environment to which it is presented (i.e., viscosity, charge, mesh size). In this regard, mucus is known to have an overall negative charge due to the high content in sialic acid (pKa 2.6) [38]. Thus, its ionization can differ in an acidic pH compared to a neutral one, and this can have an effect on the degree of interaction of mucus with the liposomal formulations, their diffusion through the mucus network, and the resulting drug permeation. Additionally, the viscosity of the hydrophilic mucus layer is known to change due to the sol-gel transition and lead to a higher viscosity at an acidic pH [12,39]. The higher mucus viscosity at pH 4.6 can, thus, impair the diffusion of the liposomal formulations through the mucus layer and, therefore, negatively affect drug permeation when compared to a pH of 7.0, as demonstrated by the low drug permeation at a pH of 4.6 for both plain and coated liposomes (Figure 2B and Figure 3B). In addition, a lower drug permeation from the coated liposomes compared to plain liposomes was found only at a pH of 4.6 (Figure 3B). The lower ibuprofen P_app_ from coated liposomes compared to plain liposomes is to be expected due to the interaction of the formulation with the mucus layer occurring as a result of the electrostatic interaction of the positively charged liposomes with the negative charge of mucus. This is due to the higher size of the coated liposomes when compared to plain liposomes (Table 3). Moreover, for chitosan-based formulations, it has been previously shown that, at a pH lower than 6, the positively charged amino groups of chitosan can electrostatically interact with negatively charged molecules, such as mucins, whereas, at a pH above 6.5, the amino groups are deprotonated and, thus, electrostatic interactions are not occurring [40].

When comparing the results obtained in the presence of mucus to the ones where both mucus and SVF were present (Figure 2C and Figure 3C, Setup 3A and B), no significant differences were found, which suggests that the presence of SVF did not have a significant impact on drug permeation for the tested drug formulations, whereas mucus was the most important element affecting the permeation of ibuprofen from the different formulations. It has been previously demonstrated that the degree of drug diffusion through a mucus layer can depend on the physicochemical characteristics of the specific drug [41]. Nonetheless, it is important to include the SVF layer, as its presence could impact the behaviour of other types of drug-delivery systems or other drugs [5]. Setup 3, which can be referred to as the vaginal-PVPA model, would, therefore, be the preferred setup when assessing drug permeability in the case of formulations destined for vaginal drug administration. The results depicted in Figure 3 highlight that the extent of drug permeation can depend on multiple factors (i.e., drug ionization, drug release, presence of a mucus layer, mucus charge, mucus viscosity, liposome-mucus interaction, etc.) and on their synergism in the specific environment. Moreover, it is evident that the pH-dependent mucus properties can critically affect the performance of liposomal formulations and the permeation of the incorporated drug. It is, therefore, highly important to include the mucus layer on top of permeation barriers to best assess the behaviour and performance of formulations destined for mucosal administration.

#### Recovery of Ibuprofen in the Donor and Acceptor Compartment

**Figure 4:**
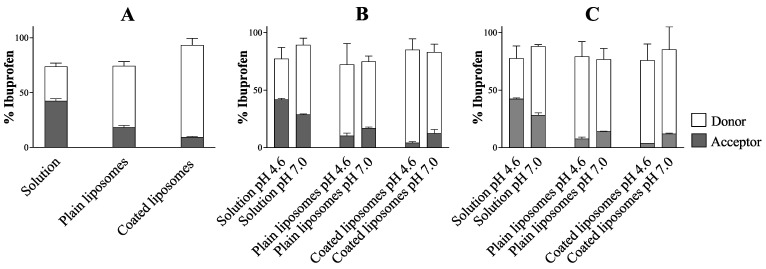
Percentage of ibuprofen recovered at the end of the permeability experiment in the donor and acceptor compartment of the Phospholipid Vesicle-based Permeation Assay (PVPA) barriers using (**A**) Setup 1 (i.e., naked barriers), (**B**) Setup 2A and B (i.e., mucus), and (**C**) Setup 3A and B (i.e., mucus + SVF) (mean ± SD, n = 18).

The amount of ibuprofen left in the donor compartment at the end of the permeation experiment was compared to the total amount permeated in the acceptor compartment to highlight how much of the initial amount of the drug was able to permeate from the different ibuprofen-loaded formulations and to evaluate the recovery of the drug. As shown in Figure 4, the percentage of the drug recovered in the donor and acceptor compartment at the end of the permeation study never reached 100%, indicating that part of the drug could be found in the PVPA barriers. In this regard, it has been previously demonstrated that drug retention in the barriers can occur for more lipophilic compounds due to their affinity for the lipidic barrier, whereas hydrophilic drugs, which have higher affinity for the aqueous donor/acceptor medium, tend to be retained to a lower extent in the PVPA barriers [42]. Therefore, due to its high lipophilicity (LogP 3.97, [27]), ibuprofen can be retained in the barriers, leading to a low recovery when comparing the amount of the drug at the start of the permeation experiment with the one found in the donor and acceptor compartment after 5 h of permeation study. The concomitant presence of ibuprofen in the acceptor compartment and inside the permeation barriers proves that all the investigated formulations would allow both systemic and local therapy.

### 3.4. Mucoadhesive Properties of the Liposomes

The mucoadhesive properties of the liposomes utilized for the delivery of ibuprofen were evaluated to further characterize the formulations and to assess if their adhesiveness to the mucus layer could be connected to the penetration potential of the drug through the permeation barriers. It has to be noted that, in order for a formulation destined for vaginal administration to be effective, it should be able to (i) be retained at the vaginal site in order to promote the (ii) release of the drug over time [43]. In order to achieve this, one of the strategies proposed to increase the resident time of the formulations in the vagina has been to prepare mucoadhesive formulations able to exploit the mucus layer covering the vaginal tissue as a docking point. The mucoadhesive properties of chitosan-based delivery systems have been gaining interest since they allow for electrostatic interaction between the cationic amino groups of chitosan and the negatively charged mucus layer [44], which, together with hydrogen bond formations and hydrophobic forces, lead to mucoadhesion [45].

**Figure 5:**
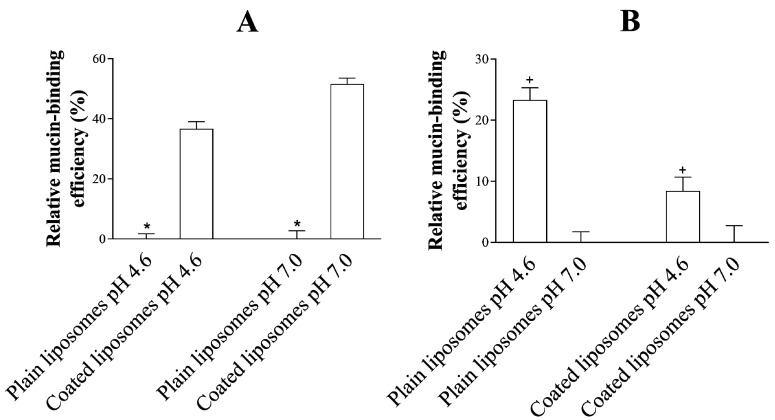
Relative mucin-binding efficiency (%) of (**A**) coated liposomes compared to plain liposomes at each pH, and (**B**) each liposomal formulation at a pH of 4.6 compared to a pH of 7.0.* Represents statistical difference between the two different liposomal formulations at the same pH. ^+^ Represents statistical difference between different pH conditions for the same liposomal formulation.

As observed in Figure 5A, the relative mucin-binding efficiency of coated liposomes (at a pH of 4.6 and 7.0) was superior when compared to the efficiency of plain liposomes, as expected and demonstrated before [13,17,19]. This evidence highlights the potential of the chitosan-based formulation in providing adhesion to mucus and prolonging their resident time *in loco*. This increased retention of the formulation inside the vagina can especially be relevant in the case of recurrent bacterial vaginosis and infections related to biofilm formation [10]. The high degree of adhesion of the coated liposomes to the mucus layer on top of the PVPA barrier can result in a lower drug permeation due to the slower diffusion of the formulation through the mucus layer and the subsequent drug release farther from the permeation barrier when compared to plain liposomes. This is clearly shown in Figure 3B,C, where the permeation of ibuprofen was lower in the case of coated liposomes when compared to plain liposomes at a pH of 4.6. Moreover, when comparing the same liposomal formulation (i.e., either plain or coated liposome, Figure 5B) at the different pH conditions investigated in this study, a lower mucin-binding effect was found in the case of a pH of 7.0 when compared to a pH of 4.6. This pH-dependent behaviour can permit a higher diffusion of the liposomal formulation through the mucus layer at a pH of 7.0 and the following higher drug permeation, as demonstrated in Figure 3B,C.

The results discussed in the last two Section 3.3 and Section 3.4 highlight the importance of considering the impact of the mucus layer on the diffusion of the liposomal formulations and the subsequent effect on drug permeation. The difference in P_app_ of ibuprofen from the two liposomal formulations discussed in Section 3.3 could be linked to the mucin-binding efficiency of the formulations, and to the pH-dependent mucus characteristics. This emphasizes the importance of having a mucus layer on top of permeation barriers aiming at mimicking mucosal tissues. According to the results obtained in this study, coated liposomes would be the preferred formulation for local treatments thanks to their lower drug permeability and higher mucin-binding, which potentially lead to enhanced residence time of the formulation on the mucosa. Moreover, this study confirmed that, when developing liposomal formulations for treating vaginal infection, or in the case of women in pre-puberty or post-menopause, it is important to consider the effect of altered pH (i.e., from pH 4.6 to pH 7.0). A change in pH could influence the mucin-binding potential of the formulation and the consequent drug permeation, which are both factors that can greatly affect the overall efficacy of the drug-delivery system.

## 4. Conclusions

In the present study, we developed a novel and reliable in vitro model able to mimic the vaginal environment and permit the assessment of drug permeation from liposomal formulations destined for vaginal administration. This model comprises both mucus and SVF, and allows for the evaluation of drug permeation at both pre-menopausal (pH 4.6) and post-menopausal/pre-puberty pH (pH 7.0) conditions. This study demonstrates that the penetration potential of the model drug ibuprofen across the vaginal-PVPA barriers was both formulation and pH-dependent and could be clearly linked to the mucoadhesion of the liposomal formulation and intrinsic characteristics of the mucus layer. As such, the vaginal-PVPA model offers a simple, reliable, and predictive permeation tool for the assessment of drug permeation from formulations destined for vaginal drug administration.

## Figures and Tables

**Table 1:** Composition of simulated vaginal fluid (SVF).

Name	Concentration (g/L)
Sodium chloride	3.510
Potassium hydroxide	1.400
Calcium hydroxide	0.222
Bovine serum albumin	0.018
Lactic acid	2.000
Acetic acid	1.000
Glycerol	0.160
Urea	0.400
Glucose	5.000
pH	4.6/7.0

**Table 2:** Setups used for the permeation studies.

Setup	SVF (10 µL)	Mucus (50 µL)
1	✕	✕
2 A: pH 4.6 B: pH 7.0	✕ ✕	✓ ✓
3 A: pH 4.6 B: pH 7.0	✓ ✓	✓ ✓

**Table 3:** Liposome characteristics: diameter, polydispersity index (PdI), and zeta potential (mean ± SD, n = 3).

Name	Diameter (nm)	PdI	Zeta Potential (mV)
Empty plain liposomes	192.6 ± 4.57	0.094 ± 0.014	−1.99 ± 3.53
Plain liposomes	193.4 ± 3.53	0.123 ± 0.020	−14.0 ± 1.87
Empty coated liposomes	203.3 ± 3.51	0.097 ± 0.015	3.06 ±. 3.87
Coated liposomes	337.9 ± 21.9	0.322 ± 0.05	42.17 ± 5.34 (Area: 50%) 64.70 ± 2.40 (Area: 50%)

## References

[1] Rossi S., Vigani B., Sandri G., Bonferoni M.C., Caramella C.M., Ferrari F., Carla C. (2019). Recent advances in the mucus-interacting approach for vaginal drug delivery: From mucoadhesive to mucus-penetrating nanoparticles. Expert Opin. Drug Deliv..

[2] Tho I., Škalko-Basnet N., Sarmento B. (2016). Cell-based in vitro models for vaginal permeability studies. Concepts and Models for Drug Permeability Studies—Cell Tissue Based in Vitro Culture Models.

[3] Vanic Z., Škalko-Basnet N., Rai M., dos Santos C.A. (2017). Nanoformulations for vaginal therapy. Nanotechnology Applied to Pharmaceutical Technology.

[4] Palmeira-De-Oliveira R., Palmeira-De-Oliveira A., De Oliveira J.M. (2015). New strategies for local treatment of vaginal infections. Adv. Drug Deliv. Rev..

[5] Das Neves J., Rocha C., Gonçalves M., Carrier R.L., Amiji M., Bahia M.F., Sarmentocde B. (2012). Interactions of Microbicide Nanoparticles with a Simulated Vaginal Fluid. Mol. Pharm..

[6] Jøraholmen M.W., Bhargava A., Julin K., Johannessen M., Skalko-Basnet N. (2020). The Antimicrobial Properties of Chitosan Can Be Tailored by Formulation. Mar. Drugs.

[7] Robinson N.B., Krieger K., Khan F.M., Huffman W., Chang M., Naik A., Yongle R., Hameed I., Krieger K., Girardi L.N. (2019). The current state of animal models in research: A review. Int. J. Surg..

[8] Grießinger J., Dünnhaupt S., Cattoz B., Griffiths P., Oh S., I Gómez S.B., Wilcox M., Pearson J., Gumbleton M., Abdulkarim M. (2015). Methods to determine the interactions of micro- and nanoparticles with mucus. Eur. J. Pharm. Biopharm..

[9] Owen D.H., Katz D.F. (1999). A vaginal fluid simulant. Contraception.

[10] Vanic Z., Rukavina Z., Manner S., Fallarero A., Uzelac L., Kralj M., Klarić D.A., Bogdanov A., Raffai T., Virok D.P. (2019). Azithromycin-liposomes as a novel approach for localized therapy of cervicovaginal bacterial infections. Int. J. Nanomed..

[11] Falavigna M., Klitgaard M., Brase C., Ternullo S., Škalko-Basnet N., Flaten G.E. (2017). Mucus-PVPA (mucus Phospholipid Vesicle-based Permeation Assay): An artificial permeability tool for drug screening and formulation development. Int. J. Pharm..

[12] Falavigna M., Klitgaard M., Steene E., Flaten G.E. (2019). Mimicking regional and fasted/fed state conditions in the intestine with the mucus-PVPA in vitro model: The impact of pH and simulated intestinal fluids on drug permeability. Eur. J. Pharm. Sci..

[13] Naderkhani E., Erber A., Škalko-Basnet N., Flaten G.E. (2014). Improved Permeability of Acyclovir: Optimization of Mucoadhesive Liposomes Using the Phospholipid Vesicle-Based Permeation Assay. J. Pharm. Sci..

[14] Cook M.T., Brown M.B. (2018). Polymeric gels for intravaginal drug delivery. J. Control. Release.

[15] Donders G., Bellen G., Grincevičienė Š., Ruban K., Vieira-Baptista P. (2017). Aerobic vaginitis: No longer a stranger. Res. Microbiol..

[16] Berginc K., Suljaković S., Skalko-Basnet N., Kristl A. (2014). Mucoadhesive liposomes as new formulation for vaginal delivery of curcumin. Eur. J. Pharm. Biopharm..

[17] Jøraholmen M.W., Vanic Z., Tho I., Skalko-Basnet N. (2014). Chitosan-coated liposomes for topical vaginal therapy: Assuring localized drug effect. Int. J. Pharm..

[18] Flaten G.E., Dhanikula A.B., Luthman K., Brandl M. (2006). Drug permeability across a phospholipid vesicle based barrier: A novel approach for studying passive diffusion. Eur. J. Pharm. Sci..

[19] Jøraholmen M.W., Skalko-Basnet N., Acharya G., Basnet P. (2015). Resveratrol-loaded liposomes for topical treatment of the vaginal inflammation and infections. Eur. J. Pharm. Sci..

[20] Berben P., Bauer-Brandl A., Brandl M., Faller B., Flaten G.E., Jacobsen A.-C., Brouwers J., Augustijns P. (2018). Drug permeability profiling using cell-free permeation tools: Overview and applications. Eur. J. Pharm. Sci..

[21] Izgü F., Bayram G., Tosun K., Izgü D. (2017). Stratum corneum lipid liposome-encapsulated panomycocin: Preparation, characterization, and the determination of antimycotic efficacy against Candida spp. isolated from patients with vulvovaginitis in an in vitro human vaginal epithelium tissue model. Int. J. Nanomed..

[22] Jøraholmen M.W., Basnet P., Tostrup M.J., Moueffaq S., Skalko-Basnet N. (2019). Localized Therapy of Vaginal Infections and Inflammation: Liposomes-In-Hydrogel Delivery System for Polyphenols. Pharmaceutics.

[23] Andersen T., Mishchenko E., Flaten G.E., Sollid J.U.E., Mattsson S., Tho I., Skalko-Basnet N. (2017). Chitosan-Based Nanomedicine to Fight Genital Candida Infections: Chitosomes. Mar. Drugs.

[24] Giordani B., Basnet P., Mishchenko E., Luppi B., Skalko-Basnet N. (2019). Utilizing Liposomal Quercetin and Gallic Acid in Localized Treatment of Vaginal Candida Infections. Pharmaceutics.

[25] Major I., McConville C. (2017). Vaginal drug delivery for the localised treatment of cervical cancer. Drug Deliv. Transl. Res..

[26] Brako F., Mahalingam S., Rami-Abraham B., Craig D.Q., Edirisinghe M. (2016). Application of nanotechnology for the development of microbicides. Nanotechnology.

[27] Benet L.Z., Broccatelli F., Oprea T.I. (2011). BDDCS Applied to Over 900 Drugs. AAPS J..

[28] Zhang H. (2016). Thin-Film Hydration Followed by Extrusion Method for Liposome Preparation. Advanced Structural Safety Studies.

[29] Mohammed A.R., Weston N., Coombes A., Fitzgerald M., Perrie Y. (2004). Liposome formulation of poorly water soluble drugs: Optimisation of drug loading and ESEM analysis of stability. Int. J. Pharm..

[30] Das Neves J., Amiji M., Sarmentocde B. (2011). Mucoadhesive nanosystems for vaginal microbicide development: Friend or foe?. Wiley Interdiscip. Rev. Nanomed. Nanobiotechnol..

[31] Andersen T., Bleher S., Flaten G.E., Tho I., Mattsson S., Skalko-Basnet N. (2015). Chitosan in Mucoadhesive Drug Delivery: Focus on Local Vaginal Therapy. Mar. Drugs.

[32] Takeuchi H., Matsui Y., Sugihara H., Yamamoto H., Kawashima Y. (2005). Effectiveness of submicron-sized, chitosan-coated liposomes in oral administration of peptide drugs. Int. J. Pharm..

[33] Naderkhani E., Isaksson J., Ryzhakov A., Flaten G.E. (2014). Development of a Biomimetic Phospholipid Vesicle-based Permeation Assay for the Estimation of Intestinal Drug Permeability. J. Pharm. Sci..

[34] Vieira A.P., Badshah S., Airoldi C. (2013). Ibuprofen-loaded chitosan and chemically modified chitosans—Release features from tablet and film forms. Int. J. Biol. Macromol..

[35] Avdeef A., Avdeef A. (2003). Charge State. Absorption and Drug Development: Solubility, Permeability, and Charge State.

[36] Eliyahu S., Almeida A., Macedo M.H., Das Neves J., Sarmentocde B., Bianco-Peled H. (2020). The effect of freeze-drying on mucoadhesion and transport of acrylated chitosan nanoparticles. Int. J. Pharm..

[37] Schattling P., Taipaleenmäki E., Zhang Y., Städler B. (2017). A Polymer Chemistry Point of View on Mucoadhesion and Mucopenetration. Macromol. Biosci..

[38] Vimr E.R., Kalivoda K.A., Deszo E.L., Steenbergen S.M. (2004). Diversity of Microbial Sialic Acid Metabolism. Microbiol. Mol. Biol. Rev..

[39] Cao X., Bansil R., Bhaskar K.R., Turner B.S., Lamont J.T., Niu N., Afdhal N. (1999). pH-dependent conformational change of gastric mucin leads to sol-gel transition. Biophys. J..

[40] Kumirska J., Weinhold M.X., Thöming J., Stepnowski P. (2011). Biomedical Activity of Chitin/Chitosan Based Materials—Influence of Physicochemical Properties Apart from Molecular Weight and Degree of N-Acetylation. Polymers.

[41] Falavigna M., Stein P.C., Flaten G.E., Di Cagno M.P. (2020). Impact of Mucin on Drug Diffusion: Development of a Straightforward In Vitro Method for the Determination of Drug Diffusivity in the Presence of Mucin. Pharmaceutics.

[42] Naderkhani E., Vasskog T., Flaten G.E. (2015). Biomimetic PVPA in vitro model for estimation of the intestinal drug permeability using fasted and fed state simulated intestinal fluids. Eur. J. Pharm. Sci..

[43] Baloglu E., Bernkop-Schnürch A., Karavana S.Y., Senyigit Z.A. (2009). Strategies to prolong the intravaginal residence time of drug delivery systems. J. Pharm. Pharm. Sci..

[44] Meng J., Sturgis T.F., Youan B.-B.C. (2011). Engineering tenofovir loaded chitosan nanoparticles to maximize microbicide mucoadhesion. Eur. J. Pharm. Sci..

[45] Rossi S., Vigani B., Bonferoni M.C., Sandri G., Caramella C., Ferrari F. (2018). Rheological analysis and mucoadhesion: A 30 year-old and still active combination. J. Pharm. Biomed. Anal..

